# Youth Depression After an Environmental Disaster: Is There a Relationship to Caregiver Stress and Depression?

**DOI:** 10.1111/cch.70058

**Published:** 2025-03-03

**Authors:** Jacqueline Dannis, Sarah Jenuwine, Kenyetta Dotson, Nicole Jones

**Affiliations:** ^1^ Charles Stewart Mott Department of Public Health Michigan State University College of Human Medicine Flint Michigan USA; ^2^ Michigan State University–Hurley Children's Hospital Pediatric Public Health Initiative Flint Michigan USA

**Keywords:** depression, environmental disaster, girls, mental health, parental stress

## Abstract

**Background:**

In the United States, female youth have been experiencing high levels of mental health challenges, including depression. Many factors increase the risk of developing mental health issues, including exposure to traumatic events, like the Flint water crisis. To better understand this connection, this study aims to answer the question: How do depression symptoms in this Flint Registry sample of girls compare with national norms and how are child depression symptoms related to parental/caregiver depression symptoms and perceived stress?

**Methods:**

The cohort included 539 girls, ages 6–17, and their parents who were Flint residents and had completed a baseline survey for their child and themselves during 2019–2021 and a follow‐up survey (2020–2022). The Behavior Assessment System for Children (BASC‐3) measured child depression symptoms; the Quality of Life in Neurological Disorders (Neuro‐QoL) depression subscale and Perceived Stress Scale Short Form (PSS‐4) measured adult depressive symptoms and perceived stress. Statistical analysis included One‐Sample Chi‐Square and Bayesian correlation and regression analysis.

**Results:**

At enrolment, the prevalence of at‐risk or clinically significant depression symptoms in Flint girls was higher than in the general population (23.6% vs. 15.9%, *p* < 0.01). At follow‐up, the prevalence decreased, yet there was still a significant difference between Flint girls (19.3%) and the general population (*p* < 0.05). Child measures of depressive symptoms at follow‐up were significantly correlated with caregiver measures of depression and stress at both time points. Child depression symptoms at baseline and caregiver depression and stress were predictive of child depressive symptoms at follow‐up.

**Conclusions:**

Results reveal high rates of depressive symptoms after a manmade environmental disaster, as well as a relationship between female youth mental wellness and caregiver well‐being. Being able to understand and identify depression symptoms in both children and caregivers is an important aspect of public health services and reinforces the call for expanding mental health screening and treatment.

**Summary**

- The prevalence of depressive symptoms is higher among youth who live in communities impacted by manmade disasters like the Flint water crisis.
- Caregiver depressive symptoms appear predictive of female youth depressive symptoms.
- There is a need for more accessible mental health services for youth and their caregivers to both diagnose and provide care.
- Continued longitudinal research is warranted.

## 1 Introduction

Female youth in the United States are experiencing pervasive mental health challenges. In 2021, 57% of female high school students reported persistent feelings of sadness or hopelessness within the past year and 30% of high school females reported having seriously considered suicide, approximately double the proportions of their male counterparts (Centers for Disease Control and Prevention 2023). The American Academy of Pediatrics (AAP) and the Office of the Surgeon General recently recognized a national paediatric mental health emergency and recommended regular screening for childhood depression at doctor's visits (AAP 2021).

Research has shown that childhood depression can be attributed to genetic factors and/or experiences of trauma, maltreatment or other early adversities (Franić et al. 2010; Samek et al. 2018). In the United States, 7% of children had at least one caregiver with poor mental health (Wolicki et al. 2021). These children are two times more likely to have mental health, behavioural health or developmental disorders compared with children with caregivers with good mental health (Thompson and Henrich 2022; Wolicki et al. 2021). A recent Harvard Graduate School of Education study reveals that depressed teens are five times more likely than teens without depression to have a depressed parent (Making Caring Common 2023).

There is also a positive relationship between parental stress and internalizing behaviours in children (Puff and Renk 2014). And parents of depressed children experience more parental stress than parents of nondepressed children (Tan and Rey 2005). This relationship often causes a cycle of increased stress among parents and increased internalizing behaviours, such as depression, in their children (Tan and Rey 2005).

The link between behavioural and mental health and traumatic manmade environmental disasters, like the Flint water crisis, has previously been shown in children (Dannis et al. 2023; Jones et al. 2022) and adults (Chung and Kim 2010; Fortenberry et al. 2018). During the Flint water crisis, 25 April 2014 to 15 October 2015, the local water supply lacked adequate corrosion control, resulting in lead and other unknown contaminants leaching into the drinking water supply. Adding to the trauma of this crisis is the acknowledgement that this was a case of environmental injustice (Flint Water Advisory Task Force 2016). Further, lead is a known neurotoxin and exposure has been associated with long‐term health effects in children, including alterations in mood and behaviour (Agency for Toxic Substances and Disease Registry 2020).

Since late 2018, the Flint Registry has been conducting survey‐based population‐level surveillance of children and families exposed to the Flint water crisis including assessments of child and caregiver depression symptoms and caregiver stress over time.

Recognizing the need to better understand the longer term mental health implications for children whose families were exposed to environmental disasters and related adversities, this study aims to answer the questions: How do depression symptoms in this Flint Registry sample of girls compare with national norms and how are child depression symptoms related to parental/caregiver depression symptoms and perceived stress?

## 2 Methods

### 2.1 Setting

This study was conducted in Flint, Michigan, after an 18‐month lead‐in‐water crisis. According to census data from 2018, one‐quarter of Flint residents were less than 18 years of age (U.S. Census Bureau 2018a). Flint residents, in general, have lower levels of education and income and higher rates of poverty when compared with State of Michigan residents (U.S. Census Bureau 2018b, 2018c). Among Flint residents over age 25, 85% have at least a high school diploma and 12% have at least a bachelor's degree (U.S. Census Bureau 2018c). With a median household income of $27 717, 40% of the Flint population and 58% of Flint children live in poverty (U.S. Census Bureau 2018b).

Before the water crisis, the City of Flint had a decades‐long population decline mainly attributed to a loss of automobile manufacturing jobs (Leiser et al. 2022). The decrease in revenue streams contributed to a financial emergency which led to the State of Michigan appointing an emergency manager to oversee the City of Flint (Leiser et al. 2022). Under this state‐appointed emergency manager, the city water source was switched from pretreated Lake Huron water provided by Detroit Water and Sewerage to locally treated Flint River water and, for over 18 months, lead leached from the drinking water infrastructure. As a result, for over 18 months, Flint citizens were exposed to unsafe drinking water with the risk of exposure to lead. They experienced significant delays in addressing their concerns about water quality, in addition to the loss of democracy at the local level (Flint Water Advisory Task Force 2016).

### 2.2 Flint Registry

In response to the water crisis, the Flint Registry, funded by the Centers for Disease Control and Prevention, is a public health registry designed to mitigate the impact of lead‐in‐water exposure and to conduct surveillance on exposed individuals. The project was built in partnership with community and incorporated community voice through multiple strategies, including working with local organizations, building a parent partners group, creating a community advisory board, holding community events, collecting feedback surveys, collaborating with a youth advisory council and seeking local community ethics review board approval (Jones et al. 2024). Community codeveloped the data collection instruments along with the project methodology. Eligibility criteria for the Flint Registry include potential exposure to the Flint water based on a connection to an address serviced by the Flint water system from 25 April 2014 to 15 October 2015, due to residency, employment, daycare or school activities. Enrolees were engaged to participate through community outreach, broad‐based marketing, city‐wide mailings and public health databases. Enrolees in the Flint Registry complete a health survey at baseline and at least 1 year later. Surveys were most often completed online and less over the telephone or in person.

### 2.3 Cohort/Sample

Included in this study are the data of 539 girls, ages 6–17, and their parents who were Flint residents at the time of the water crisis and completed a baseline survey for their child and themselves in 2019 through 2021 and a follow‐up survey for their child in 2020 through 2022. This represents about 30% of the 1803 Flint Registry female child participants who were residents of Flint during the water crisis who completed a baseline survey and were eligible to complete the follow‐up survey.

The child surveys were completed by mothers 92.9% and fathers 7.1% of the time. The average duration between the completion of the baseline and follow‐up surveys was 16.1 months ± 5.5 months.

The parents reported that the girls were Flint residents during the water crisis and whether the children drank unfiltered Flint tap water during the Flint water crisis. The identities of the child and adult participants were verified with Michigan Department of Health and Human Services records.

### 2.4 Measures/Outcomes

#### 2.4.1 Childhood Depressive Symptoms

Parent‐reported depression symptoms in girls were measured with the Behavior Assessment System for Children, Third Edition (BASC‐3) (Reynolds and Kamphaus 2015). The BASC‐3 Parent Rating Scale is a rigorous, nationally normed measure of adaptive and problem behaviours in community and home settings (Reynolds and Kamphaus 2015). It underwent bias analyses, was age standardized and has norms derived from a large, representative sample of children across the United States (Reynolds and Kamphaus 2015). The BASC‐3 was chosen to measure depressive symptoms because it is a strong, oft‐used clinical measure of parent report of childhood behaviour.

The BASC‐3 depression scale measures common symptoms of depression, including sudden changes in mood, negativity and sadness that may result in the inability to carry out everyday activities. The scale asks parents to report the frequency of child behaviour in the last several months such as ‘Cries easily’ with four response categories from Never to Almost Always.

The depression scale includes 13 items used to calculate composite age‐standardized *T*‐scores. Clinically relevant normative categories—very low, low, average, at‐risk and clinically significant—are derived from the standard *T*‐score distribution with a mean of 50 and standard deviation of 10.

The BASC‐3 manual reports the reliability, Cronbach's alpha, of the depression scale as 0.86 for female school‐age children and ranges from 0.90 to 0.95 for female adolescents (Reynolds and Kamphaus 2015). In the current study, Cronbach's alphas were 0.89 at baseline and 0.91 at follow‐up for school‐age children and 0.92 at baseline and follow‐up for adolescents.

#### 2.4.2 Adult Depressive Symptoms

Depression symptoms in parents were measured with the Quality of Life in Neurological Disorders (Neuro‐QoL) depression subscale (Cella et al. 2012). Neuro‐QoL instruments were developed to be psychometrically sound and clinically relevant measurement tools for individuals with neurological conditions or disorders.

The Neuro‐QoL depression 8‐item short form measures common symptoms of depression including feelings of hopelessness, negative mood and decreased positive affect. The scale includes items such as ‘In the past 7 days I felt hopeless’ with five response categories from Never to Always.

Raw scores were calculated and converted to a standardized *T*‐score distribution with a mean of 50 and a standard deviation of 10. The standardized scale was developed using a reference sample of the US general population.

The reported reliability, Cronbach's alpha, of the depression scale is 0.96 (Cella et al. 2012). For the current study, the Cronbach's alpha of the depression scale is 0.96 for both the baseline and follow‐up measures.

#### 2.4.3 Caregiver Stress

Parental stress was measured using the Perceived Stress Scale 4‐Item Short Form (PSS‐4) (Cohen et al. 1983), a 4‐item scale designed to measure adult perception of stress. The scale includes items such as ‘In the last month, how often have you felt that you were unable to control the important things in your life’ with five response categories from Never to Very Often.

Raw scores for each item are used to calculate a scaled score from 0 to 16. The established norm mean perceived stress score for the general population is 4.49 with a standard deviation of 2.96 and reliability of 0.6 (Cohen and Williamson 1988). In the current study, Cronbach's alpha was 0.61 for the baseline and 0.63 for the follow‐up measures.

#### 2.4.4 Demographics

Child age, race/ethnicity and utilization of free and reduced‐cost meal services were also reported.

### 2.5 Statistical Analysis

The survey data were collected using REDCap electronic data capture tools hosted at Michigan State University (Harris et al. 2009). SPSS version 28 was employed to analyse the data using One‐Sample Chi‐Square and Bayesian correlation and regression analysis.

Chi‐Square analysis was performed to compare the prevalence of Flint Registry girls' depression symptoms with national norms (Reynolds and Kamphaus 2015).

Bayesian regression analysis was employed to model the relationship between girls' depression symptoms and parent depression symptoms and stress. Child depression at baseline and parent depression and stress at enrolment and follow‐up were included in the model to predict child depression at follow‐up. The JZS method was used to test model versus the null model, and the *F* test for model fit was reported.

There were less than 3% missing cases for the demographic variables (see Table 1). Missing data for the child depression measure was 5.2% at baseline and 9.6% at follow‐up. Missing data for the adult variables of depression was 4.1% at baseline and 21.0% at follow‐up and stress was 2.0% at baseline and 19.5% at follow‐up. Most of the missing adult depression and stress data at follow‐up, 18.9%, can be attributed to parents who did not complete the follow‐up survey.

To address missing data, imputation and Bayesian statistics were employed (Enders 2022). With less than 5% missing data for the child depression measures, multiple imputation was performed using all child demographic and scaled measures before Chi‐Square analysis comparing the prevalence of depression symptoms of Flint Registry girls to the BASC‐3 national derived norms (reference procedure in SPSS) (Jakobsen et al. 2017). SPSS missing value analysis revealed no patterns in missing data. With about 20% of missing data for the adult depression and stress measures at follow‐up, Bayesian statistics without imputation were employed to address missing data in the correlation and regression analysis.

### 2.6 Ethics Statement

This study was conducted after the Michigan State University Institutional Review Board approved the secondary analysis of Flint Registry data and in accordance with federal, state and local regulations, university policies and ethical standards.

## 3 Results

The average age of the 539 girls at enrolment was 10 ± 2.9 years. Eighty‐four percent were eligible for free or reduced‐cost meals at school. Most parents reported that their child identified as Black or African American only (67.7%), followed by White only (21.9%) and Hispanic only (1.1%). Eight percent identified their child as more than one race or ethnicity. The parents reported that the girls were Flint residents during the water crisis and 84.8% of children drank unfiltered Flint tap water during the Flint water crisis (Table 1).

**TABLE 1:** Flint children at enrolment (December 2018–March 2020) (*N* = 539).

Child study participant demographics	Mean (SD) or percent
Age	10 (2.9)
Free or reduced‐cost meals	
No	14.3%
Yes	84.7%
Missing	2.0%
Ethnicity	
African American only	67.7%
European American only	21.9%
Middle Eastern only	0.0%
Hispanic only	1.1%
Asian only	0.0%
Native American only	0.2%
More than one ethnicity	8.0%
Missing	1.1%
Exposure to unfiltered water for drinking	
Every day	72.0%
Less than every day	12.8%
Not at all	10.0%
Missing	5.2%

Abbreviation: SD, standard deviation.

The means and standard deviations for the child depression scales and the parent depression and stress scales at baseline and follow‐up are provided in Table 2.

**TABLE 2:** Means and standard deviations: Child depression symptoms and parent depression symptoms and perceived stress (*N* = 539).

	*N*	Mean	Standard deviation
Child BASC‐3 depression symptoms at baseline	510	52.71	12.81
Child BASC‐3 depression symptoms at follow‐up	487	50.22	10.56
Parent Neuro‐QoL depression symptoms at baseline	517	48.84	9.38
Parent Neuro‐QoL depression symptoms at follow‐up	426	48.01	9.14
Parent PSS‐4 perceived stress at baseline	528	6.67	3.10
Parent PSS‐4 perceived stress at follow‐up	434	6.53	3.16

Abbreviations: BASC‐3, Behavior Assessment System for Children, Third Edition; Neuro‐QoL, Quality of Life in Neurological Disorders; PSS‐4, Perceived Stress Scale 4‐Item Short Form.

The prevalence of at‐risk or clinically significant depression symptoms was significantly higher in Flint Registry girls at baseline than expected in the BASC‐3 norms from a general population: 23.6% versus 15.9% [*X* ^2^ (1, *N* = 539) = 23.9256, *p* < 0.01]. The prevalence of depression symptoms decreased at follow‐up though remained significantly higher than expected in the general population, 19.3% versus 15.9% [*X* ^2^ (1, *N* = 539) = 4.9244, *p* < 0.05].

Child measures of depression at follow‐up were significantly correlated with child measure of depressive symptoms at baseline and caregiver measures of depressive symptoms and stress at baseline and follow‐up, as shown in Table 3. Analysis showed no impact of socioeconomic status, as measured by free or reduced lunch eligibility, on child depression measures at follow‐up.

**TABLE 3:** Bayesian pairwise Pearson correlations: Child depression symptoms and parent depression symptoms and perceived stress.

	*N*	Child BASC‐3 depression symptoms at follow‐up
Child BASC‐3 depression symptoms at baseline	463	0.638**
Parent Neuro‐QoL depression symptoms at follow‐up	388	0.378**
Parent Neuro‐QoL depression symptoms at baseline	468	0.319**
Parent PSS‐4 perceived stress at follow‐up	396	0.261**
Parent PSS‐4 perceived stress at baseline	478	0.200**

Abbreviations: BASC‐3, Behavior Assessment System for Children, Third Edition; Neuro‐QoL, Quality of Life in Neurological Disorders; PSS‐4, Perceived Stress Scale 4‐Item Short Form.

** *p* < 0.01.

Child depression at baseline and caregiver depression and stress at baseline and follow‐up were predictive of child depression at follow‐up, with a good model fit [*F* (5,345) = 55.211, *p <* 0.01], and explain about 45% of the variance in child depression at follow‐up (*R* ^2^ = 0.444) (Table 4). Child depression symptoms at baseline and caregiver depression symptoms at follow‐up were the strongest predictors of child depression symptoms.

**TABLE 4:** Bayes regression model fit analysis.

*F* test	df	*R*	*R* square	Adjusted *R* square	Std. error of the estimate
55.211**	(5345)	0.667	0.444	0.436	7.9597

** *p* < 0.01.

## 4 Discussion

In this large cohort of female youth, the findings reveal a high rate of depression symptoms after being exposed to a water crisis. This study mirrors research that demonstrates the long‐term impacts of the Flint water crisis on child behavioural and mental health outcomes (Cuthbertson et al. 2016; Dannis et al. 2023; Ezell and Chase 2021; Jones et al. 2022; Sneed et al. 2020). These findings also reflect the lived experiences of Flint Registry participants. When asked to describe the impact of the Flint water crisis, one member of the Flint Registry Parent Partner Advisory group highlighted the long‐term influence on their mental health, ‘Being the caregiver of children affected by the Flint water crisis goes beyond the physical health concerns, it affected our mental safety as well. In 2014, my children were 2 and 4 years old. Given this man‐made disaster we were robbed the rights to a normal childhood for them’ (A. Strozier, personal communication, 2024).

The data were collected 4–6 years after the end of the crisis; however, the findings are consistent with other studies of the Flint water crisis, which have found an impact on mental and behavioural health (Cuthbertson et al. 2016; Ezell and Chase 2021; Sneed et al. 2020), along with other work that demonstrates the long‐term psychological effects of community‐level trauma (Adams and Boscarino 2005; Pfefferbaum et al. 1999). The data trend found here with a slight decrease in parent‐reported child symptoms in 2021 is shown with national surveys (NCHS Data Query System 2024). Further, national surveys show little difference in parent‐reported depression symptoms of children living in large versus medium and small metropolitan areas like Flint (NCHS Data Query System 2024).

Another limitation of this study is that the registry is a voluntary surveillance tool. Yet, significant efforts were made to recruit participants through community‐engaged and noncommunity‐engaged methods (Jones et al. 2024). In addition, survey collection was offered in multiple modes to reduce barriers to participation. The strength of the study lies in that the sample size is large, and data were collected at two time points, baseline and follow‐up, on average 16 months after the baseline survey.

There is some potential good news in the data. Overall, the depression symptoms of the Flint Registry girls did not worsen with time and appeared to alleviate for some. This could be due to the intensive efforts of initiatives like the Flint Registry to engage children in mental health and other support services.

Like the recent Caring for Caregivers report (Making Caring Common 2023), these findings reveal a strong relationship between female youth and caregiver well‐being and emphasize the importance of (1) educational programs to support parents in identifying and understanding the signs of depression and (2) combined parent and child interventions, such as family therapy, to break the cycle of depression.

The data also add to the evidence of the need to expand early mental health screening and treatment for mental health disorders, both in general and in partnership with the local schools, where interventions have been shown to increase receipt of health care (Dittus et al. 2014). Based on their findings that half of children with depression continue to experience some impairment in daily functioning in early adulthood, Powell et al. (2023) call for more longitudinal studies of depressive symptoms to support children and their caregivers through the life span. Considering the Flint Registry will continue to follow the children and families exposed to the Flint water crisis, the current study provides a first step towards answering this call.

## Author Contributions

**Jacqueline Dannis:** conceptualization, data curation, formal analysis, methodology, supervision, writing – original draft, writing – review and editing. **Sarah Jenuwine:** conceptualization, data curation, formal analysis, writing – original draft, writing – review and editing. **Kenyetta Dotson:** investigation, writing – review and editing. **Nicole Jones:** funding acquisition, project administration, supervision, writing – original draft, writing – review and editing.

## Ethics Statement

This study was conducted after the Michigan State University Institutional Review Board approved the secondary analysis of Flint Registry data and in accordance with federal, state and local regulations, university policies and ethical standards.

## Consent

Adult participants provided informed consent (written, digital or over the phone) for themselves and their children to enrol in the Flint Registry.

## Conflicts of Interest

The authors declare no conflicts of interest.

## Data Availability

Requested data may be provided after IRB approval and appropriate data use agreements have been obtained.
